# Saunders’ Hand Atlas

**Published:** 1903-10

**Authors:** 


SAUNDERS' HAND ATLAS.—Atlas and Epitome of Operative Surgery. By Dr. Otto Zuckerkandl, Privat-docent in the University of Vienna. Second edition, revised and enlarged. Authorized translation from the German. Edited by J. Chalmers DaCosta, M. D., Professor of the Principles of Surgery and of Clinical Surgery in Jefferson Medical College, Philadelphia; Surgeon to the Philadelphia Hospital, etc. With forty colored plates and 278 illustrations in the text. W. B. Saunders & Co., Philadelphia and London. Price, $3.00 net.

In this work Dr. Zuckerkandl outlines in a comprehensive and easily understood manner the rules and methods employed by the best surgeons of the day in practically all important surgical procedures. His descriptions are reinforced by a large number of well selected and excellently executed original cuts. The work is intended chiefly for the use of students and surgeons of limited experience. The author says "those groups of operations whose practice upon the cadaver forms the basis of practical instruction, are described in detail and illustrated in their most conspicuous aspects. Other operations, whose performance falls largely to the lot of the skilled surgeon, and whose practice upon the cadaver appears less important, are described concisely." Altogether the text is excellent and has been ably edited by Dr. DaCosta. However, the illustrations alone are worth many times the cost of the entire volume.

